# Continuous process technology for glucoside production from sucrose using a whole cell-derived solid catalyst of sucrose phosphorylase

**DOI:** 10.1007/s00253-021-11411-x

**Published:** 2021-06-30

**Authors:** Andreas Kruschitz, Linda Peinsipp, Martin Pfeiffer, Bernd Nidetzky

**Affiliations:** 1 Austrian Centre of Industrial Biotechnology (acib), Krenngasse 37, 8010 Graz, Austria; 2 Institute of Biotechnology and Biochemical Engineering, Graz University of Technology, NAWI Graz, Petersgasse 12, 8010 Graz, Austria

**Keywords:** Continuous biomanufacturing, Flow bio-catalysis, Whole cell-based enzyme immobilization, Packed-bed reactor, Sucrose phosphorylase, 2-α-d-Glucosyl-glycerol

## Abstract

**Abstract:**

Advanced biotransformation processes typically involve the upstream processing part performed continuously and interlinked tightly with the product isolation. Key in their development is a catalyst that is highly active, operationally robust, conveniently produced, and recyclable. A promising strategy to obtain such catalyst is to encapsulate enzymes as permeabilized whole cells in porous polymer materials. Here, we show immobilization of the sucrose phosphorylase from *Bifidobacterium adolescentis* (P134Q-variant) by encapsulating the corresponding *E. coli* cells into polyacrylamide. Applying the solid catalyst, we demonstrate continuous production of the commercial extremolyte 2-α-d-glucosyl-glycerol (2-GG) from sucrose and glycerol. The solid catalyst exhibited similar activity (≥70%) as the cell-free extract (~800 U g^−1^ cell wet weight) and showed excellent in-operando stability (40 °C) over 6 weeks in a packed-bed reactor. Systematic study of immobilization parameters related to catalyst activity led to the identification of cell loading and catalyst particle size as important factors of process optimization. Using glycerol in excess (1.8 M), we analyzed sucrose conversion dependent on space velocity (0.075–0.750 h^−1^) and revealed conditions for full conversion of up to 900 mM sucrose. The maximum 2-GG space-time yield reached was 45 g L^−1^ h^−1^ for a product concentration of 120 g L^−1^. Collectively, our study establishes a step-economic route towards a practical whole cell-derived solid catalyst of sucrose phosphorylase, enabling continuous production of glucosides from sucrose. This strengthens the current biomanufacturing of 2-GG, but also has significant replication potential for other sucrose-derived glucosides, promoting their industrial scale production using sucrose phosphorylase.

**Key points:**

• *Cells of sucrose phosphorylase fixed in polyacrylamide were highly active and stable.*

• *Solid catalyst was integrated with continuous flow to reach high process efficiency.*

• *Generic process technology to efficiently produce glucosides from sucrose is shown.*

**Graphical abstract:**

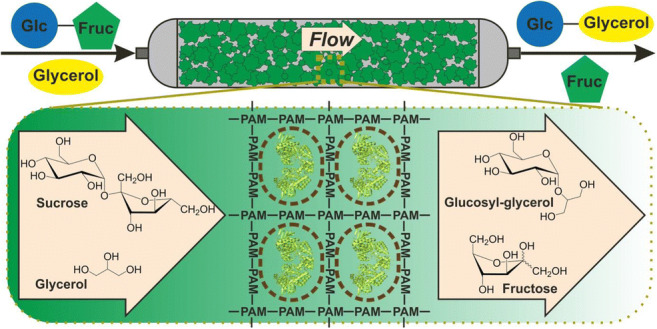

**Supplementary Information:**

The online version contains supplementary material available at 10.1007/s00253-021-11411-x.

## Introduction

Advanced biomanufacturing targets the industrial implementation of innovative process technologies for a radically improved bioproduction (Sheldon and Woodley 2018; Woodley 2020; Wu et al. 2021). It builds on the idea of continuous bioprocessing, especially in the upstream part of the process, and involves process intensification and integration as key guiding principles (Tamborini et al. 2018; De Santis et al. 2020). Continuous bioprocessing with enzymes for chemical synthesis can be viewed as realization of the trendsetting idea of “flow bio-catalysis” in industrial applications (Buchholz et al. 2012; Thompson et al. 2019; Santi et al. 2021). Biotransformation performed continuously promotes the tight interconnection of the upstream and downstream parts of the production process for optimum overall output of product (Liese et al. 2006; Tamborini et al. 2018). Moreover, it facilitates the implementation of process control strategies to ensure consistency, quality and efficiency (Gargalo et al. 2020).

Central pillar in the integrated structure of any continuous process is the catalyst (Hartman 2020). Considering catalyst design, physical separation of the catalyst from the product is the primary criterion. By way of this separation, facile recycling of the catalyst should be enabled in the particular reactor configuration envisaged (e.g., packed-bed reactor) (Wachtmeister and Rother 2016; Pinto et al. 2020). With the product dissolved in the bulk fluid, the catalyst is usually obtained as a solid preparation of the enzymes used. “Heterogenization” of the homogenous catalyst (the soluble enzyme) involves some form of immobilization (Di Cosimo et al. 2013; Liese and Hilterhaus 2013; Guisan et al. 2020). From the broad selection of methods known, only a tiny portion is promising to meet the challenging requirements that industrial biomanufacturing processes typically involve. A suitable balance of key characteristics is demanded of the immobilized enzyme catalyst: high specific activity per total solid mass; general robustness as regards enzyme activity and structural-mechanical stability under in-operando conditions; flexible use across operation scales; and efficient production in a step-economic procedure (Basso and Serban 2019). Encapsulation of permeabilized whole cells in a porous polymer matrix is interesting, offering just that balance in a unique fashion (Zajkoska et al. 2013).

Enzyme immobilization by way of whole cell encapsulation is long known for industrial catalyst preparation. Glucose isomerase represents a classic example of immobilized enzyme application in large-scale bioprocessing (Bhosale et al. 1996). Nitrilase (Hann et al. 2002) and aspartase (Chibata et al. 1986) are also important examples. In the current era of recombinant enzyme production in high-performance expression hosts (Liu et al. 2013), whole cell encapsulation comes into focus again. The obtained cells effectively represent “nanobioreactors” that can concentrate the desired enzyme activity to the physical limit of solubility (≥100 g L^−1^) of the functional protein. Encapsulated cells, in principle, support continuous processing in all basic types of bioreactor, irrespective of the fluidics and back mixing characteristics of the used apparatus (Pinto et al. 2020). A number of enzymes have been immobilized via encapsulation of whole cells of the native microorganism. Among them was sucrose phosphorylase (SucP) (Vandamme et al. 1987) which is also the target of the current inquiry, performed in the actual context of industrial biomanufacturing of the cosmetic ingredient 2-α-d-glucosyl-glycerol (2-GG) (Goedl et al. 2008; Luley-Goedl et al. 2010; Tan et al. 2016; Roenneke et al. 2018; Zhang et al. 2020).

The upstream part of the 2-GG process involves SucP-catalyzed trans-glycosylation of sucrose (donor) to glycerol (acceptor), as shown in Scheme S1. 2-GG and fructose are the products (Goedl et al. 2008; Luley-Goedl et al. 2010). Of note, the product downstream processing can involve nanofiltration (Kruschitz and Nidetzky 2020a), optionally combined with reactive extraction (Kruschitz and Nidetzky 2020b). The biotransformation is performed with glycerol in excess (≥1.5-fold) over sucrose, to minimize donor hydrolysis resulting in glucose release (Scheme S1) (Goedl et al. 2008). As shown recently, a mechanism-based kinetic model of the overall reaction is a powerful engineering tool to optimize the 2-GG synthesis for different processing tasks (Klimacek et al. 2020). With 2-GG concentrations of ~1.0 M (254 g L^−1^) reached and substrates used at their combined solubility limit, efforts at further process intensification shift from the biotransformation as such to the actual mode of process operation. Thus, continuous processing comes strongly into focus for 2-GG production. This in turn places special emphasis on the SucP catalyst used. The 2-GG synthesis was reported with different preparations of recombinant SucP (i.e., soluble enzyme in different purity grades; carrier-bound immobilized enzyme; cross-linked enzyme aggregates) (Goedl et al. 2008; De Winter et al. 2012). SucP immobilization was additionally investigated in porous carriers of different solid material and surface groups (Goedl et al. 2007; Cerdobbel et al. 2010; De Winter et al. 2011; Bolivar et al. 2017). Early studies showed enzyme from the natural source (e.g., *Leuconostoc mesenteroides*) in whole cell-immobilized form to produce glucose 1-phosphate from sucrose in continuous reaction. P_2_O_5_-dried or gelatin-encapsulated cells were used, but production of the *L. mesenteroides* derived catalyst required careful optimization and showed low volumetric productivity (Vandamme et al. 1987). Collectively, the previous approaches of SucP immobilization were not fully convincing in the sum of the abovementioned criteria of a successful catalyst for industrial biomanufacturing. General drawback of immobilization via surface tethering was the extra processing required to capture the soluble SucP in partially purified form (Goedl et al. 2007; Cerdobbel et al. 2010; Bolivar et al. 2017). We also noted the possibility of attaching whole cells onto solid carriers, but the aggregate view from literature suggests this to be a less promising route of enzyme immobilization (Zajkoska et al. 2013; Guisan et al. 2020).

Heterologous expression in *Escherichia coli* is efficient means of recombinant production of SucP. Here, we therefore explored polymer encapsulation of whole *E. coli* cells, with the aim of developing a competitive SucP catalyst for continuous production of 2-GG. We also considered the substantial replication potential of a continuous process technology for biocatalytic glycosylation from sucrose. A number of α-d-glucoside products are accessible via SucP-catalyzed glycosylation (Goedl et al. 2010; Franceus and Desmet 2020), several of which (e.g., kojibiose (Beerens et al. 2017)) have considerable significance for industrial production. Having in mind material properties of the cell-polymer composite important for continuous bioprocessing (e.g., swelling, hardness, elasticity), we examined two principal routes of cell encapsulation. One was based on an ionotropic hydrogel, the other used a synthetically polymerized matrix. We show that cell encapsulation in polyacrylamide (PAM) fulfilled the demand for balanced characteristics of activity, robustness, and facile preparation of the solid SucP catalyst. Systematic study revealed critical parameters of an efficient catalyst preparation. Using the catalyst thus obtained, we demonstrate continuous production of 2-GG in a packed-bed reactor that was operated stably at excellent output parameters over weeks.

## Materials and methods

### Chemicals

Acrylamide (97%), 3-(dimethylamino)propionitrile (98%), and glutaraldehyde were from Sigma-Aldrich (St. Louis, Missouri, USA); N,N’-methylenebis(acrylamide) was from Carl Roth (Karlsruhe, Germany); potassium persulfate (99%) was from Merck (Darmstadt, Germany); κ-carrageenan was from Biosynth Carbosynth (Berkshire, UK); and 1,6-diaminohexane was from Fluka (Buchs, Switzerland). Glycoin® natural, containing 52.8 wt.% 2-GG, was from bitop AG (Dortmund, Germany). All other chemicals (at least of reagent grade) were from Carl Roth, Merck, or Honeywell (Charlotte, North Carolina, USA).

### Cell cultivation

*E. coli* BL21 (DE3)-*agp* harboring pQE30 plasmid was used to produce SucP from *L. mesenteroides* (*Lm*SucP, N-terminal His-tag) (Goedl et al. 2007) and a SucP variant from *Bifidobacterium adolescentis* (*Ba*SucP_P134Q, N-terminal His-tag) (Franceus et al. 2021). The feature of deleted *agp* gene (α-glucose 1-phosphate phosphatase) in the *E. coli* strain used can be useful when working with glycoside phosphorylases, but it was not essential here. Reported protocols were used for cultivation in shake flasks (Goedl et al. 2007) or in a 5-L bioreactor (Unterweger et al. 2012). Pre-cultures (50 mL in 300-mL baffled flasks for shake flask cultivation, 200 mL in 1000-mL baffled flasks for bioreactor cultivation), incubated in LB-medium (10 g L^−1^ peptone, 5 g L^−1^ yeast extract, and 5 g L^−1^ NaCl) overnight at 30 °C and 130 rpm, were used to inoculate the main culture. Cell production in shake flasks (1 L in 2-L baffled flasks) was carried out in LB medium, containing 100 mg L^−1^ ampicillin, at 37 °C and 110 rpm (GFL 3033 incubator; GFL, Burgwedel, Germany). After induction (0.25 mM IPTG) at an OD of 0.8–1.0, cultivation continued overnight at 25 °C and 110 rpm. Bioreactor cultivation was done at 37 °C with a Biostat® CT (5 L) system (B. Braun Biotech International, Germany) equipped with a Biostat® C controller (Unterweger et al. 2012). Table S1 and Table S2 show the medium used. After induction (0.25 mM IPTG) at an OD of 2.0–3.0, cultivation was done overnight at 25 °C. The pH was maintained at 7 with 2 M KOH or 1 M H_3_PO_4_. The air flow was 7.5 L min^−1^ and the stirrer speed was adjusted (150–730 rpm) automatically for 40% air saturation. 5 mL Ampicillin (115 mg mL^−1^) was added twice, before inoculation and induction. Cells were harvested with a HiCen SR ultracentrifuge (Herolab, Wiesloch, Germany) at 4 °C and a RCF of 4400*g* for 20 min. The cell pellets were frozen at −21 °C to permeabilize the cells.

### Catalyst preparation

When referring to whole cells, we mean the *E. coli* cells obtained by a single freeze-thaw cycle. Suspension of whole cells, cell-free extract, and encapsulated whole cells were compared as enzyme catalyst. The cell suspension used thawed cells (0.1 g mL^−1^) in 100 mM HEPES buffer (pH 7.0). Cell-free extract was prepared from cell suspension by three times ultra-sonification (Sonde micropointe 3.1 mm, Thermo Fisher, Waltham, MA, USA) for 6 min (2 s pulse on, 4 s pulse off) with 30% amplitude. Cell debris were removed by ultra-centrifugation (Eppendorf 5424 R, Eppendorf AG, Hamburg, Germany) at 4 °C and a RCF of 21,130*g* for 45 min (Schwaiger et al. 2020). Encapsulation of whole cells was done in κ-carrageenan (Tosa et al. 1979) or PAM (Tosa et al. 1974; Yamamoto et al. 1974). κ-Carrageenan was dissolved in pre-heated MilliQ water or 0.9% NaCl at 0.02–0.03 g mL^−1^. The solution was stirred at 300 rpm and 50 °C on a MR 3001 K magnetic stirring hotplate (Heidolph Instruments, Schwabach, Germany). The whole cells (0.5 g wet cells mL^−1^) in 10 mM HEPES buffer (pH 7.0) or 0.9% NaCl were vortexed (REAX top, Heidolph Instruments) and mixed with the κ-carrageenan solution at a 2:3 ratio (by volume) at 500 rpm for 10 s. The suspension was put on ice for 15 min to cure. Resulting gel was soaked in 0.3 M KCl for 30 min, cut into small pieces with a scalpel, and washed with deionized water and 0.3 M KCl. For hardening, the particles were suspended (at a ratio of 0.5 g mL^−1^) in 0.5 M phosphate buffer, containing 0.3 M KCl and 85 mM 1,6-diaminohexane, and incubated on ice for 10 min. Afterwards, 25% glutaraldehyde solution was added to 3 vol.% and the mixture stirred at 150 rpm for 30 min on ice. Finally, the particles were washed with 0.3 M KCl.

**Fig. 1:**
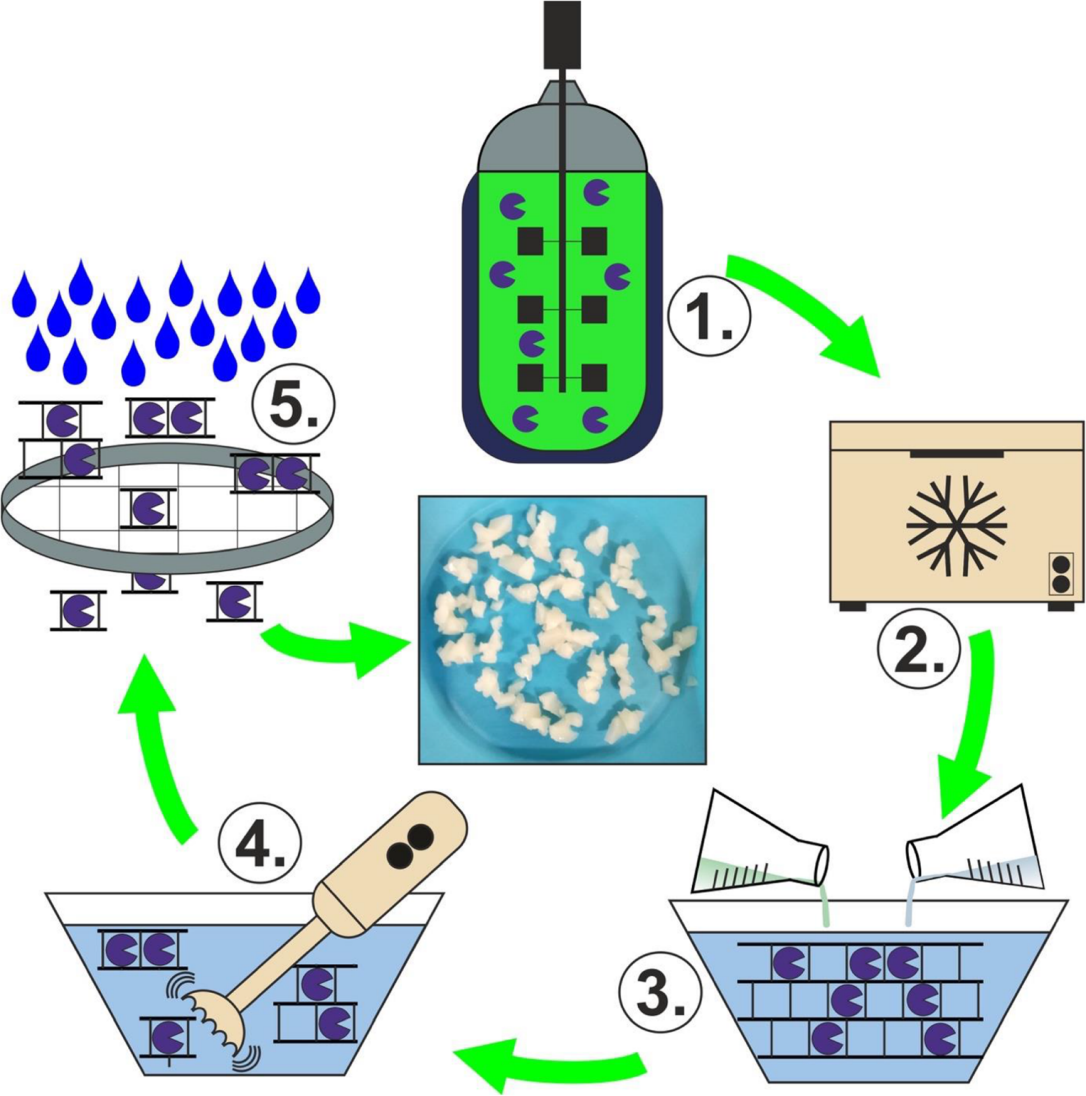
Work-flow for enzyme catalyst preparation via whole cell encapsulation in PAM. (1) Cell production in the bioreactor, (2) cell permeabilization by freeze-thaw treatment, (3) encapsulation, (4) shredding, and (5) sieving plus washing. In the middle, a photograph of the final PAM-I particles is shown

For encapsulation in PAM, whole cells were resuspended in 100 mM HEPES buffer (pH 7.0). The suspension (4–25 mL) was thoroughly mixed in a glass beaker at 300 rpm to provide a homogeneous cell suspension. The cell loading was varied between 0.25 and 1.00 g wet cells per mL cell suspension (for a cell loading of 1 g mL^−1^, wet cells were used undiluted). Acrylamide was dissolved directly into the stirred cell suspension at concentrations between 0.0625 and 0.4375 g mL^−1^. N,N’-Methylenebis(acrylamide) and 3-(dimethylamino)propionitrile solution (5 vol.%) were admixed as cross-linking agent and polymerization accelerator, respectively. The suspension was permanently stirred at 300 rpm. Polymerization was started with 2.5 wt.% potassium persulfate solution. Cross-linking agent, polymerization accelerator, and initiator were added at 10 mg, 0.125 mL, and 0.125 mL per mL cell suspension, respectively. Polymerization started within a minute and was completed after 30 min. The entire polymerization process was performed in an ice bath to counter the arising heat of polymerization and to prevent a possible damage of the microbial cells (Jack and Zajic 2005). The obtained rigid PAM material (PAM-I) was first cut with a scalpel and subsequently shredded with a hand blender for some seconds. The resulting PAM-I particles were sieved (mesh sizes of 0.25–2.00 mm) to control the particle size and afterwards thoroughly washed with deionized water on the sieves. The whole process is depicted in Fig. 1. Particle properties, including consistency, shape, swelling, and compression, were optically monitored.

### Reaction in batch

Encapsulated whole cells were compared with suspended whole cells and cell-free extract. The substrate solution contained 300 mM sucrose and 1.8 M glycerol. Reactions were standardized on the same equivalent of wet cells added (around 5 mg). The size of the immobilized enzyme particles was 1–2 mm. Reactions were performed in 2 mL for 120 min at 30 °C and a 900-rpm agitation rate on a Thermomixer comfort (Eppendorf AG). The influence of cell loading, acrylamide concentration, and temperature (30–50 °C) on 2-GG production using the PAM-I particles was also investigated in batch reactions. Sucrose (300–350 mM) and the six-fold amount of glycerol were used. Reactions’ conditions were the same as above. About 50 mg of PAM-I particles were added to each 2-mL reaction solutions. Samples were taken at certain times, heat-treated at 99 °C for 5 min, and analyzed by HPLC.

### Continuous reaction in a packed-bed reactor

PAM-I particles (size range in mm: 0.25–1.00, 1.00–2.00, and >2.00) were packed by hand into XK26/20 columns (ID 26 mm, GE Healthcare, Chicago, IL, USA) and thoroughly flushed with deionized water. Unless stated, the bed (i.e., reactor) volume was 40 mL. The column temperature was controlled at 40 °C from a circulating water bath connected to the column’s jacket. A Smartline Pump 1000 or an Azura P 4.1S (Knauer, Berlin, Germany) delivered feed from the substrate solution. The feed flow rate and the composition of the substrate varied as indicated in the results. Samples were taken at reactor outlet and analyzed by HPLC. The continuous reactor was used to assess the in-operando stability of the enzyme catalyst and to analyze reaction performance in dependence of operational parameters (e.g., flow rate). For determining stability, continuous reaction was performed (with downtimes in-between) until the product release had decreased to below half of the initial value. During the downtimes, the reactor was stored in substrate solution at ~22 °C.

### Analytics

HPLC analysis was done on a Shimadzu LC-20AD (Kyoto, Japan) or a Merck Hitachi L-7100 (Darmstadt, Germany) system. Both were equipped with a autosampler (SIL-20AC HT ➔ Shimadzu, L-7250 ➔ Merck) and a refractive index detector (RID-20A ➔ Shimadzu, L-7490 ➔ Merck). A YMC-Pack Polyamine II/S-5 μm/12 nm column (250×4.6 mm) (YMC, Kyoto, Japan) (Holtkamp et al. 2009) with or without a guard column (20×4 mm) was used. Isocratic elution used acetonitrile/water (75/25, by volume). Twenty-microliter sample was injected. HPLC was operated at a flow rate of 1 mL min^−1^ at 25 °C. The run time was 30 min. All components of the reaction (sucrose, 2-GG and the corresponding 1-*O*-regioisomer, fructose, and glucose) were separated, as shown in Figure S1, and quantified. The glycerol was also separated, but due to its presence in excess, it was not used for quantification. Reaction selectivity was calculated on a mole basis as [2-GG]/[Fructose] (see Equation 2 in Supplementary Information).

## Results

### Encapsulation for preparation of whole cell-based SucP catalyst

*E. coli* cells containing *Lm*SucP or *Ba*SucP_P134Q were encapsulated in κ-carrageenan and PAM. *Lm*SucP has been in use for 2-GG production for several years (Goedl et al. 2008). The enzyme exhibits ~95% regioselectivity for the desired 2-*O*-regioisomer of α-glucosyl glycerol, yet its stability at envisaged process temperatures (≥40 °C) is rather low. The *Ba*SucP is more stable but less regioselective (~65%) than *Lm*SucP. The *Ba*SucP_P134Q variant was developed (Franceus et al. 2021) to confer regioselectivity (83±3%) to the stable enzyme. Measured in cell-free extract (*N* = 2) for 30 min using ~0.3 M sucrose and ~1.8 M glycerol, the specific activity at 30 °C of *Lm*SucP and *Ba*SucP_P134Q was 792±86 and 794±57 U g^−1^ cell wet weight, respectively. κ-Carrageenan and PAM were proposed for whole cell encapsulation in earlier studies (Tosa et al. 1974; Nishida et al. 1979; Trelles et al. 2004; Zajkoska et al. 2013). We here assessed the two encapsulation strategies for preparation of a whole cell-based SucP catalyst suitable for continuous production of 2-GG in a packed-bed enzyme reactor. In terms of activity and stability, the catalyst was expected to fulfill the earlier mentioned requirements of industrial usability.

Reproducible encapsulation in κ-carrageenan proved difficult, primarily because gelation was extremely temperature sensitive and thus hard to control (Hann et al. 2002). Besides material inhomogeneity noted, the κ-carrageenan material dissolved within minutes in the applied substrate solution (0.3 M sucrose, 1.8 M glycerol). Cross-linking with glutaraldehyde improved the material’s chemical stability, yet considerable amount of enzyme activity of both *Lm*SucP and *Ba*SucP_P134Q was lost from the material within a week.

In contrast to κ-carrageenan, PAM enabled cell encapsulation with far better control. Due to the consciously triggered polymerization of the PAM, reproducible production of a homogeneous material (PAM-I) was ensured. The PAM-I was rigid and thus easy to shred. Particles were stable in substrate solution. Their consistency and shape were unchanged under use in agitated suspension or in a packed-bed reactor. Swelling was not observed. PAM encapsulation of *Lm*SucP cells (cell loading, 0.5 g mL^−1^; acrylamide, 0.1875 g mL^−1^) showed high activity (60–70% of cell extract), but the in-operando stability at 30 °C of a packed-bed enzyme reactor (25 mL; particles ≥2 mm) was rather low (half-life, ~12 days; Figure S2). Considering that systematic study of *Lm*SucP stabilization in encapsulated whole cells would be a challenging task, we turned our attention to *Ba*SucP_P134Q as a supposedly more robust catalyst. Results reported later were all obtained with *Ba*SucP_P134Q.

**Fig. 2:**
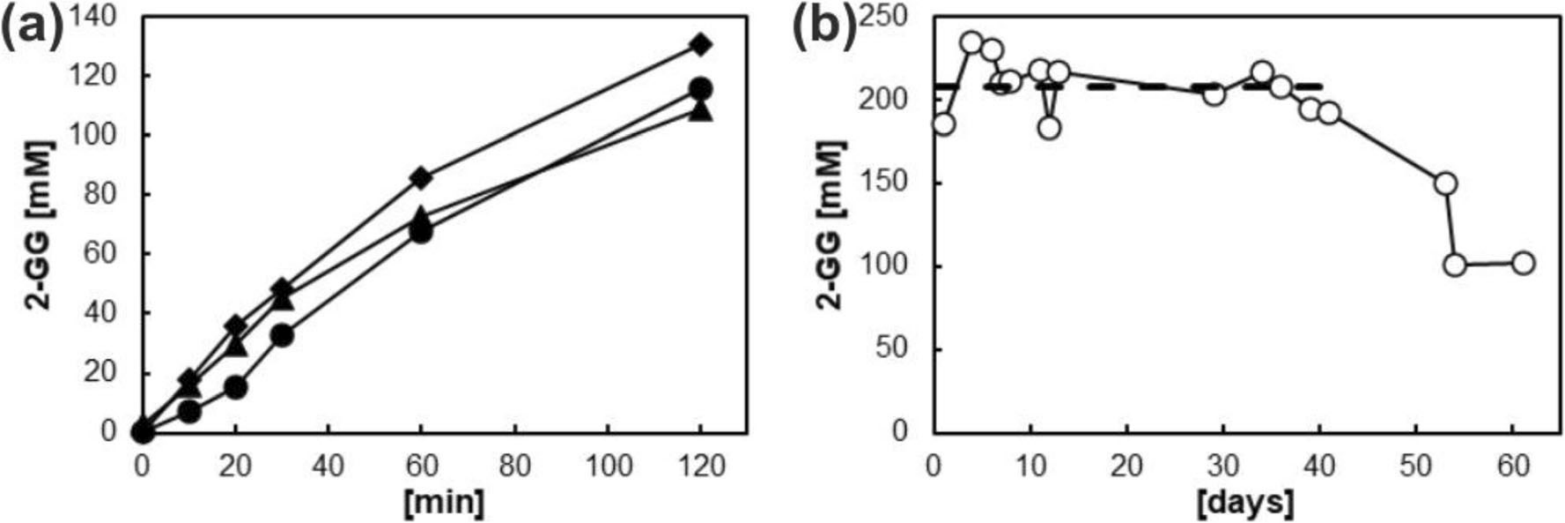
Activity (**a**) and in-operando stability (**b**) of PAM-encapsulated whole cells of *E. coli* expressing *Ba*SucP_P134Q. **a** Batch reaction at 30 °C using 307 mM sucrose and 1.8 M glycerol to release 2-GG and fructose (not shown). PAM-I particles (cell loading, 0.5 g mL^−**1**^; acrylamide, 0.1875 g mL^−**1**^; size, 1–2 mm) (●), suspended whole cells (▲), and cell-free extract (♦) were used. Reactions were standardized on the equivalent of 5 mg well cells added. **b** Continuous reaction at 40 °C using a packed-bed reactor (40 mL) of PAM-I particles (size, ≥2 mm). The space velocity was 0.15 h^−**1**^, and 300–350 mM sucrose and 1.8 M glycerol were used. The 2-GG concentration (○) in the effluent is shown. The dashed line is the mean 2-GG concentration in the effluent within the first 40 days. The PAM-I particles used were prepared with a cell loading of 0.25 g mL^−**1**^ and acrylamide concentration of 0.1875 g mL^−**1**^

PAM-I particles (1-2 mm) were examined in batch synthesis of 2-GG using the suspended whole cells and the cell extract for reference (Fig. 2a). The encapsulated cells retained most (≥70%) of the activity of the other two enzyme preparations, and their regioselectivity for glycosylating the glycerol O2 compared to the O1 was identical (85–90%). Likewise, the overall 2-GG selectivity (2-GG produced per fructose released; Equation 2 in the Supplementary Information) was identical (83±3%). The bigger sized fraction of particles (≥2 mm) was assembled into a packed-bed (40 mL) and used in continuous reaction. The 2-GG release at steady state was stable at 208 (±16) mM for ~40 days, only to decrease later as shown in Fig. 2b. The overall (apparent) half-life of the catalyst was estimated as ~55 days. Of note, conversion of the limiting sucrose substrate (≤80%) was below the maximum. These results imply, therefore, that the 2-GG formation in the time of up to ~40 days was controlled by factor(s) other than the enzyme activity present in the PAM-I particles. We discuss later the likely involvement of external mass transfer as a rate-limiting factor (Liese and Hilterhaus 2013).

### Tailoring the properties of the catalytic PAM-I material

**Fig. 3:**
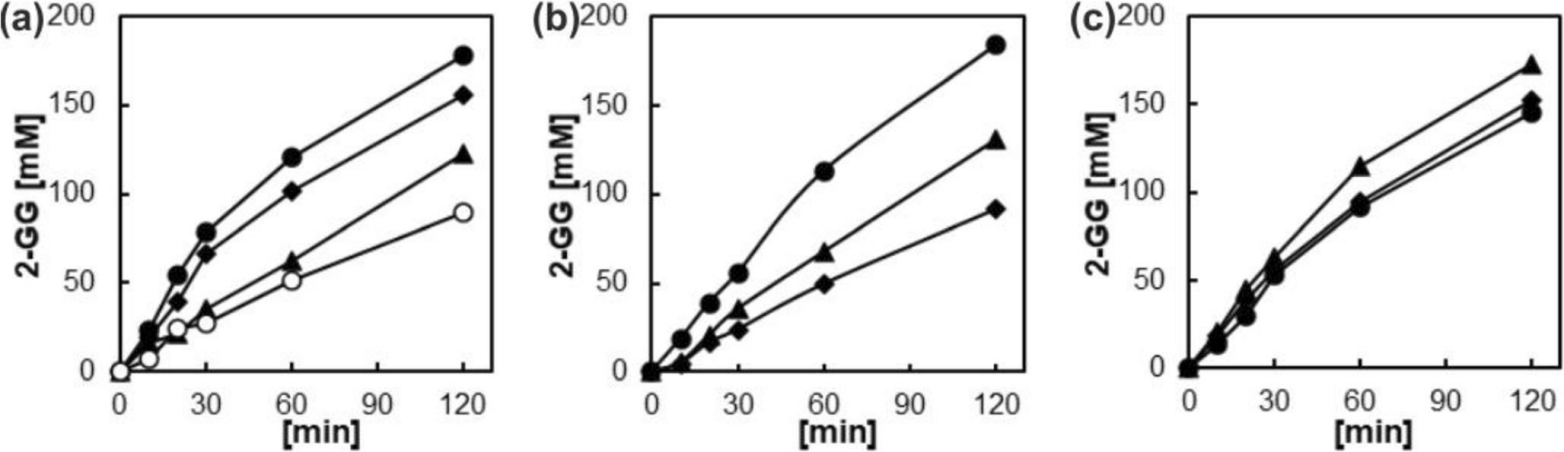
Characterization of catalytic PAM-I particles obtained under varied encapsulation conditions. **a** Cell loading varied at 0.25 (○), 0.50 (▲), 0.75 (♦), and 1.00 (●) g wet cells mL^−**1**^. Other conditions: sucrose, 316 ± 8 mM; glycerol, 1.8 M; acrylamide, 0.1875 g mL^−**1**^; and 30 °C. **b** Acrylamide varied at 0.1875 (●), 0.3125 (▲), and 0.4375 (♦) g mL^−**1**^. Other conditions: sucrose, 348 ± 11 mM; glycerol, 1.8 M; cell loading, 0.5 g mL^−**1**^; and 30 °C. **c** Temperature varied at 30 (●), 40 (▲), and 50 °C (♦). Other conditions: sucrose, 302 ± 2 mM; cell loading, 0.5 g mL^−**1**^; and acrylamide, 0.1875 g mL^−**1**^

Optimization of the catalytic PAM-I material required balance between specific activity (U g^−1^ solid mass) and final consistency of the solid particles. We examined cell suspensions with loadings in the range 0.25–1.00 g mL^−1^ and acrylamide concentrations in the range 0.0625–0.4375 g mL^−1^. Cell loading benefited the specific activity as expected (Fig. 3a), but we noted that at loadings of ≥0.75 g mL^−1^ the PAM polymerization became spatially inhomogeneous due to difficult mixing. Moreover, the effectiveness of solid catalyst (the degree to which the loaded activity is expressed in the observable reaction rate) decreased as the cell loading increased (Fig 3a). Likewise, the acrylamide concentration was crucial for material strength, with polymerization at 0.0625 g mL^−1^ yielding only a soft sludge. However, the PAM material also got increasingly brittle as higher acrylamide concentrations were used. Material processability into mm-sized particles restrained the acrylamide concentration to the upper limit used.

Figure 3a, b shows assessment of the different PAM-I particles in batch synthesis of 2-GG. Normalized on solid mass added, the PAM-I particles of highest cell loading also gave the highest 2-GG release rates (Fig. 3a). However, fourfold cell loading only resulted in twofold increase in rate. The decrease in enzyme effectiveness thus implied a diffusional limitation of the 2-GG rate, already reported for other encapsulated cells (Cheetham et al. 1985; Trelles et al. 2004; McIver et al. 2008). “Overloading” the PAM-I material with cells was considered to be potentially useful, however, to extend the in-operando lifetime of the solid catalyst in continuous reaction. Compromise between efficient use of cells, catalyst stability and consistent material preparation was to apply a cell suspension with a loading of 0.5 g mL^−1^ (Fig. 3a).

The acrylamide concentration affected the catalyst activity negatively (Fig. 3b). Tighter material structure obtained at higher acrylamide loading could further restrict the mass transfer into and out of the solid catalyst, as shown for a different cell-matrix system (Carballeira et al. 2009). Moreover, high acrylamide could damage the cells during polymerization, as suggested from earlier work (Fukui and Tanaka 1982). Accordingly, we chose 0.1875 g mL^−1^ for further studies.

Temperature in the range 30–50 °C had a very small effect on the 2-GG release rate, with 40 °C being best (Fig. 3c). The free enzyme (cell extract) exhibited a ~30% increase in rate due to the same temperature increase. Diffusion is generally less temperature dependent than enzyme catalysis. The results therefore further support the suggestion of mass transfer controlling the reaction rate of the solid catalyst. The 2-GG selectivity (84±4%) was unaffected by the used variations in temperature, cell loading and acrylamide dosage.

### Continuous 2-GG production in a packed-bed reactor

**Fig. 4:**
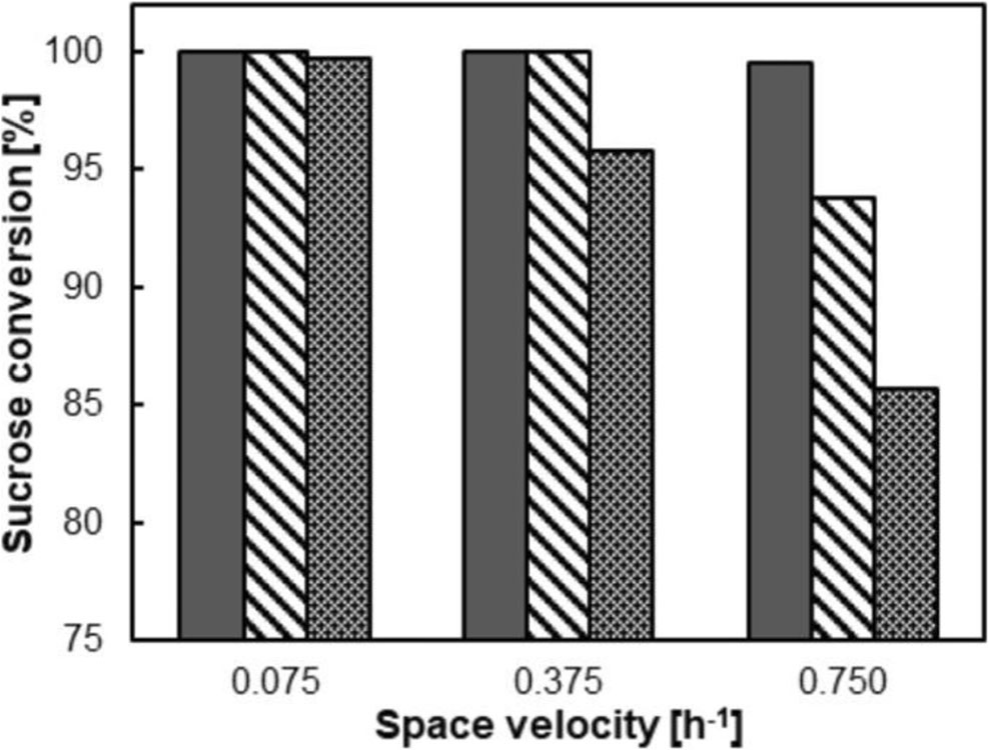
Sucrose conversion in packed-bed reactor operated at three different space velocities. The packed-bed reactor was filled with PAM-I particles in the size range of 0.25–1.00 (dark gray), 1.00–2.00 (diagonal stripes), or >2 mm (dotted), respectively. A sucrose concentration of 326 ± 8 mm and 40 °C were used

The PAM-I catalyst is usable flexibly in different reactor formats. However, we were interested in the packed-bed reactor (Figure S3) as it involves an industrially proven concept for continuous reaction applicable throughout all scales (Pinto et al. 2020). Due to the high catalyst concentration in the bed volume, the reactor is ideal to pursue process intensification. The PAM-I particles were sieved into size fractions (0.25–1.00; 1.00–2.00; ≥2.00 mm) that were used separately (Figure S4). Particle size variation was considered for its aggregate effect on external and internal mass transfer. Space velocity was also varied in a tenfold range. Results are summarized in Table 1 and Fig. 4. To analyze the data, we defined as processing task that the applied sucrose (~300 mM) should be converted to ≥99%. Considering 2-GG synthesis integrated with product purification, the task reflects a real process need. Reported procedures of downstream processing (Kruschitz and Nidetzky 2020a; Kruschitz and Nidetzky 2020b) are incapable of separating sucrose and 2-GG. The trends of the data that conversion decreased with increasing space velocity and that the effect was stronger with bigger-sized particles were consistent with expectations from chemical engineering theory. Only the smallest particle fraction, giving the most efficient mass transfer, enabled the processing task to be reached up until the highest space velocity used. However, uneven packing of bed, leading to visible channeling and by-passing of liquid flow (Figure S4a), was a problem when working with these particles. Steady operation of the continuous reactor for longer periods can be quite difficult under these conditions. Therefore, to still employ the small particles for practical effect, we combined the small with the intermediate particle fraction. An exemplary particle size distribution and microscopic images of the PAM-I particles can be found in Figure S5.

**Table 1:** Continuous production of 2-GG in packed-bed reactor with space velocity (SV) adapted to sucrose conversion task

Sucrose [mM]	*SV* [h^−1^]	2-GG [g L^−1^]^a^ (χ)^b^	*STY* [g L^−1^ h^−1^]^c^ (η)^d^
296 ± 3	0.750	61 ± 1 (100%)	45.8 (14%)
596 ± 4	0.375	119 ± 1 (99%)	44.5 (14%)
879 ± 40	0.240 0.180	144 ± 10 (86%) 171 ± 1 (94%)	34.6 (11%) 30.7 (9%)

Conditions: PAM-I particles (0.25–2.00 mm), wet cell loading of the packed-bed volume (40 mL) of 0.21 g mL^−1^, 40 °C

^a^2-GG titer in the effluent of the packed-bed reactor, 2-GG selectivity was identical (83±1%)

^b^Conversion χ of sucrose reached

^c^Space-time yield achieved in the packed-bed reactor (Eq. 3 in Supplementary Information)

^d^Catalytic effectiveness η of the catalyst compared to cell extract (see Eq. 4 in Supplementary Information)

Using the reactor bed assembled from the blended particle material, we analyzed 2-GG production from sucrose supplied at varied concentrations (300–900 mM). We considered that increase in the sucrose loading could be industrially relevant not only by its obvious effect on the 2-GG concentration attainable, but also by benefiting the degree of utilization of glycerol. The glycerol was supplied at a constant concentration of 1.8 M, sufficient to shut down hydrolysis of sucrose which is a known side reaction (<6% of activity of 2-GG synthesis) catalyzed by the enzyme (Scheme S1). The space velocity was adjusted to demonstrate sucrose conversion at, or close to, the challenging processing task. Results are summarized in Table 1. Importantly, the overall 2-GG selectivity was constant (83±1%) under all conditions used. Despite the high solute concentration used (total substrate, 470 g L^−1^), the packed-bed stayed unaltered (e.g., no compression) during operation and the distribution of the liquid flow appeared homogeneous, with no evidence of channeling seen. The PAM-I particles retained their shape and consistency. Swelling was not observed. The resulting pressure drop across the reactor was ≤2 bar, which may need to be considered for a potential scale up.

The sucrose conversion was complete (≥99%) for the low and intermediate concentrations, while it was between 86 and 94% for the highest concentration, depending on the space velocity used. There was consistent relationship between 2-GG concentration (twofold increase) and space-time yield (*STY*; no change) when the sucrose concentration was doubled (296 mM → 596 mM) and the space velocity was decreased twofold accordingly (0.750 h^−1^ → 0.375 h^−1^). Results with 900 mM sucrose no longer followed the trend that a proportional decrease in space velocity could compensate the effect of increase in the sucrose concentration. Both the 2-GG concentration and the *STY* were decreased (Table 1). Plausible reason for the lowered conversion rate at 900 mM sucrose was that the enhanced micro-viscosity of the fluid at high sucrose loading affected the efficiency of mass transfer processes.

Mass transfer effect on the conversion rate was further implied by the effectiveness factor (η) of the solid catalyst. The η was determined with Equation 4 (Supplementary Information) from the known cell loading of the packed-bed volume (0.21 g mL^−1^) using the conversion rate (for ~99.5% sucrose conversion) of the cell extract in batch conversion (7.8 mmol h^−1^ g^−1^) as the reference. The value of η (Table 1) from the flow reactor experiment was considerably lower than the relative activity (≥70%) obtained from the agitated reaction in batch (Fig. 2a). This suggested external mass transport (film diffusion to the solid surface) in the flow reactor as major rate-limiting factor. For the long-term operation (Fig. 2b), this implies that in the stable production region, 2-GG synthesis was controlled by diffusion. From the point on where 2-GG concentration in the effluent decreased, the loss of initial biocatalytic activity was considerable and thus enzyme activity became the limiting factor of 2-GG synthesis.

## Discussion

As development of an enzymatic transformation proceeds from exploration to implementation, decision on the catalyst preparation used is of high priority. It is made in the knowledge of the precedent-setting significance of the catalyst preparation for the process technology used in production. Considering biomanufacturing of 2-GG at multi-ton per year production scale (Luley-Goedl et al. 2010), we here addressed the key issue of developing a sucrose phosphorylase catalyst suitable for continuous processing at an industrially competitive performance. Based on the evidence shown, we propose PAM encapsulation of whole *E. coli* cells harboring the recombinant *Ba*SucP_P134Q.

### Encapsulation method

To select polymer material for encapsulation, we considered thermogels (e.g., agar), ionic hydrogels (e.g., κ-carrageenan), and organic synthetic matrices (e.g., PAM, polyvinyl alcohol). LentiKat® encapsulation technology is based on polyvinyl alcohol. Due to the upper limit of usable temperature in the range 30–40 °C (Zajkoska et al. 2013), thermogels were excluded and the more stable ionic hydrogels used instead. Facile preparation and flexibly tuned properties of the catalytic material, without need for specialized (pre)industrial equipment of fabrication, were main reasons for choosing PAM instead of polyvinyl alcohol. However, LentiKats® are not rejected at this stage. Their uniform shape and size are tailored for high mass transport and easy separation from the reaction medium, all of which makes them interesting for continuous operation in flow reactors (Krasňan et al. 2016). But LentiKats® production is time consuming and requires dedicated equipment such as the LentiPrinter® (Rebroš et al. 2013; Krasňan et al. 2018). Therefore, for now only the PAM succeeded in meeting our set aim of a straightforward encapsulation process. Of note, the application of PAM is considered as safe, provided that residual acrylamide is negligible (Anderson 2005; Nyyssölä and Ahlgren 2019). PAM is approved in various industrial sectors, including the cosmetic industry (Gaytán et al. 2021).

Results reveal PAM as much better qualified for 2-GG production than κ-carrageenan. The PAM encapsulation was controllable and reproducible, as required for repeated production of homogeneous PAM-I material. The whole process took less than an hour, saving time (≥14-fold) compared to covalent enzyme immobilization (*Lm*SucP: Goedl et al. 2007; *Ba*SucP: Cerdobbel et al. 2010) as well as to LentiKat® encapsulation in general (Rebroš et al. 2013). Before application, it was necessary to tailor properties, including size and the associated surface area, of the PAM-I. Shredding and sieving were used to process the PAM-I material into catalytic particles (size ≥0.25 mm) suitable in all respects of activity and stability for continuous conversion in a packed-bed reactor. Both methods are amenable for material processing and particle fabrication throughout operation scales. Particle size and shape could still be relevant factors for further optimization of the catalyst, to improve the external mass transfer for reaction intensification.

### Enzyme selection

Enzyme comparison by way of the corresponding *E. coli* cells fabricated into PAM-I particles showed *Ba*SucP_P134Q to be more promising for application than *Lm*SucP. Only to note, both enzymes were expressed in *E. coli* to similar levels of activity (~800 U g^−1^ cell wet weight). Examined under in-operando conditions of a packed-bed reactor producing 2-GG, the approximate half-life of the *Lm*SucP at 30 °C (~12 days; Figure S2) was lower than the half-life of *Ba*SucP_P134Q at 40 °C (~55 days) estimated from the decline phase in activity in Fig. 2b. The 2-GG selectivity of the enzymes was between 80 and 85%. Overall, therefore, when assessed as whole cell catalyst for 2-GG production, the P134Q variant of *Ba*SucP served the purpose of a selective and stable catalyst, thus strongly supporting the antecedent molecular engineering of the enzyme (Franceus et al. 2021).

### Continuous process technology

Operation of the packed-bed reactor, containing the tailored PAM-I particles, was practical. The sieving ensured the removal of very small particles which could potentially clog the reactor. The bed showed no alteration during continuous operation, indicating that a constant flow pattern was provided. In the case of a scale-up of the packed-bed reactor, however, the bed properties must be thoroughly monitored for an increase of the reactor dimensions (i.e., inner diameter, height) might influence the bed. The continuous bioconversion also enables the feasible integration of the upstream part with the downstream part to form a holistic process line. A separation of the biocatalyst from the product solution is obsolete. The established process technology has also high replication potential and could be used beyond the production of 2-GG. It could enable the production of several other sucrose-derived glucosides (e.g., nigerose (Kraus et al. 2016), kojibiose (Beerens et al. 2017)) and facilitate the transition from lab-scale to industrial-scale production.

### Performance comparison

The achieved in-operando stability (~6 weeks) in the packed-bed reactor is promising for an industrial application. It was higher than for other functional carbohydrates (i.e., tagatose) production processes using immobilized cells (~3 weeks) (Jung et al. 2005). It was also increased by five- to six-fold compared to the stability obtained for the continuous production of 2-GG with immobilized *Lm*SucP (Bolivar et al. 2017). The in-operando stability test, with a stable 2-GG production over 40 days (Fig. 2b), revealed a total turnover number (*TTN*, Equation 5) of around 65 g 2-GG g^−1^ cell wet weight. For the best operation conditions found (assuming a PAM-I in-operando stability of at least 40 days), a *TTN* of more than 200 g 2-GG g^−1^ cell wet weight could be calculated. This is an excellent value for a fine chemical product (Liese et al. 2006; Tufvesson et al. 2011; Wu et al. 2021). Operation of the packed-bed reactor was also possible at high substrate loadings (up to 300 g L^−1^ sucrose), delivering excellent process productivities (Table 1) (Straathof 2014; De Santis et al. 2020). The calculated *STY* was higher than the highest glucosyl-glycerol productivity (24.3 g L^−1^ h^−1^) reported by now, which was achieved with a coupled phosphorolysis and trans-glycosylation two-enzyme system (Zhang et al. 2020). The achieved glucosyl-glycerol titer in the Zhang et al.’s study was higher (452 g L^−1^) than that reached here. However, direct comparison of our study with that of Zhang et al. (2020) is difficult, since different enzyme systems, biocatalyst formulations, and reaction set-ups were applied. The productivities achieved with the packed-bed reactor were also higher than for comparable processes using immobilized cells. For example, the *STY* of acetic acid production in a fluidized-bed reactor or of l-lactic acid production in a fed-batch reactor was below 10 g L^−1^ h^−1^ (Yamane and Tanaka 2013; Straathof 2014). Chemo-enzymatic production of glycolic acid (a skin care product) in a CSTR reached *STY* between 20 and 30 g L^−1^ h^−1^ (Panova et al. 2007). The established continuous 2-GG production process with PAM-I performs also well in comparison with industrial processes that apply immobilized whole cells. A prominent example is the industrial production of l-aspartic acid from fumaric acid which was developed nearly half a century ago. Whole cells of l-aspartase encapsulated in κ-carrageenan or PAM could be applied in packed-bed reactors with volumes up to 1000 L. An estimated l-aspartic acid throughput of around 140 kg h^−1^ could thereby be achieved (Chibata 1996). DuPont (Wilmington, USA) applied nitrilase-containing whole cells in alginate to produce 4-cyanopentanoic acid, a precursor in the production of the commercialized cleaning solvent 1,5-dimethyl-2-piperidone. *STY* between 50 and 80 g L^−1^ h^−1^ and an outstanding *TTN* of up to 3.5 kg g^−1^ cell dry weight were achieved in batch conversion (Hann et al. 2002). The production of isomaltulose (i.e., palatinose) is an example of the sugar industry. It is produced from sucrose using immobilized whole cells of glucosyl-transferase. *STY* up to 40 g L^−1^ h^−1^ with product concentrations of around 200 g L^−1^ were achieved in a packed-bed reactor (Cheetham 1987).

In conclusion, the herein developed continuous process technology shows considerable potential for an intensified 2-GG production at industrial scale. Furthermore, the process technology can be generically applied for production of other sucrose-derived trans-glycosylation products.

## Supplementary Information

ESM 1 (PDF 9924 kb)

## Data Availability

Data obtained in the current study are available from the DOI 10.5281/zenodo.4663666.
