# Supplementary material for: Continuous process technology for glucoside production from sucrose using a whole cell-derived solid catalyst of sucrose phosphorylase
Source: Appl Microbiol Biotechnol. 2021 Jun 30;105(13):5383–94. doi: 10.1007/s00253-021-11411-x (PMC8285329; doi:10.1007/s00253-021-11411-x)
Supplement: Supplementary file 1 — (PDF 9924 kb) [file 253_2021_11411_MOESM1_ESM.pdf]

Supplementary Information

Applied Microbiology and Biotechnology

Continuous process technology for glucoside production from sucrose using a whole cell-derived solid catalyst of sucrose phosphorylase

Andreas KRUSCHITZ[1,2], Linda PEINSIPP[1,2], Martin PFEIFFER[1,2] and Bernd NIDETZKY[1,2,*]

[1]Austrian Centre of Industrial Biotechnology (acib), Krenngasse 37, 8010 Graz. Austria

[2]Institute of Biotechnology and Biochemical Engineering, Graz University of Technology, NAWI Graz, Petersgasse 12, 8010 Graz. Austria

*Corresponding author, E-mail: bernd.nidetzky@tugraz.at; phone: +433168738400; fax: +433168738434

Reaction scheme

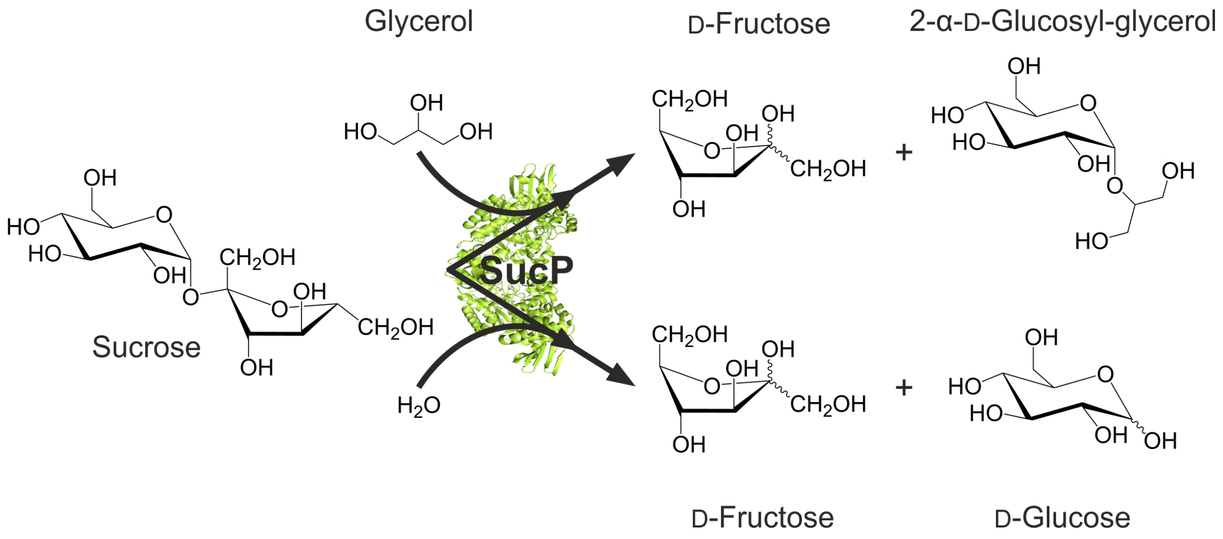

Scheme S1 Reaction scheme for the synthesis of 2-α-D-glucosyl-glycerol (2-GG) by sucrose phosphorylase (SucP). 2-GG and D-fructose are formed as main products. Sucrose hydrolysis which is a side reaction catalyzed by SucP is also shown (Klimacek et al. 2020)

Cell cultivation

Table S1: Chemical composition for 4.5 L of fermentation medium

Compound	Quantity
Glucose monohydrate	110 g
Peptone from Casein	50 g
Yeast extract	25 g
NaCl	25 g
KH_2PO_4	20.75 g
K_2HPO_4	17 g
NH_4Cl	5 g
$MgSO_4 \cdot 7H_2O$	1.25 g
Trace element solution (see Table S2)	5 mL
Polypropylene glycol	0.5 mL

Table S2: Composition of the trace element solution

Compound	Concentration
$FeSO_4 \cdot 7H_2O$	4 g L^{-1}
$MnSO_4 \cdot H_2O$	1 g L^{-1}
$AlCl_3 \cdot 6H_2O$	0.55 g L^{-1}
$CoCl_2$	0.4 g L^{-1}
$ZnSO_4 \cdot 7H_2O$	0.2 g L^{-1}
$Na_2MoO_4 \cdot 2H_2O$	0.2 g L^{-1}
$CuSO_4 \cdot 5H_2O$	0.15 g L^{-1}
H_3BO_3	0.1 g L^{-1}

Performance parameters

The space velocity (SV), the 2-α-D-glucosyl-glycerol selectivity (S_{2-GG}, mole basis), the space-time yield (STY), the effectiveness factor (η) and the total turnover number (TTN) were defined as:

$$SV = \frac{\dot{v}}{V} = \frac{1}{\tau} \quad (1)$$

$$S_{2-GG} = \frac{c_{2-GG}}{c_{Fructose}} \quad (2)$$

$$STY = SV \cdot c_{2-GG} \cdot MW_{2-GG} \quad (3)$$

$$\eta = \frac{\frac{V_{PBR} \cdot c_{Sucrose,PBR} \cdot \chi_{PBR}}{\tau_{PBR} \cdot m_{PBR}}}{\frac{V_{BC} \cdot c_{Sucrose,BC} \cdot \chi_{BC}}{\tau_{BC} \cdot m_{BC}}} \quad (4)$$

$$TTN = \frac{SV \cdot V \cdot t \cdot c_{2-GG} \cdot MW_{2-GG}}{m} \quad (5)$$

$\dot{v}$ was the flow rate, V was the reactor volume, τ was the residence time, c was the molar concentration, MW was the molecular weight, χ was the sucrose conversion m was the mass of applied wet cells and t was the operation time in hours. The subscripts $2-GG$, BC and PBR stand for 2-α-D-glucosyl-glycerol, batch conversion and packed bed reaction, respectively. The 2-GG selectivity (S_{2-GG}) was calculated based on the molar amount of fructose formed, which represents the highest amount of 2-GG that can be theoretically formed. SucP also catalyzes the formation of glucose (due to sucrose hydrolysis, Scheme S1) and of the isomer 1-α-D-glucosyl-glycerol (Goedl et al. 2008; Franceus et al. 2021). Sucrose hydrolysis was measured to be <6% of the activity of 2-GG synthesis. The 2-GG regioselectivity ([2-GG]/([2-GG]+[1-GG]) was measured to be 85-90%. In total, the 2-GG yield is thus lowered compared to fructose. The effectiveness factor η was calculated as the ratio of the conversion rate achieved with the PAM-I in the packed bed reactor and the conversion rate achieved with the cell free extract in the batch reactions. For the batch reaction the conversion rate was calculated for 99.5% sucrose conversion χ_{BC}. Batch reactions were performed with 2 mL substrate solution, comprising a sucrose concentration of 300 mM, and 50 µL of cell extract (0.1 g wet cells mL^{-1}). The residence time τ_{BC} to achieve the targeted conversion was estimated to be ~15.7 h. Parameters for the conversion rate achieved with the PAM-I in the packed bed reactor can be found in Table 1.

Analytics

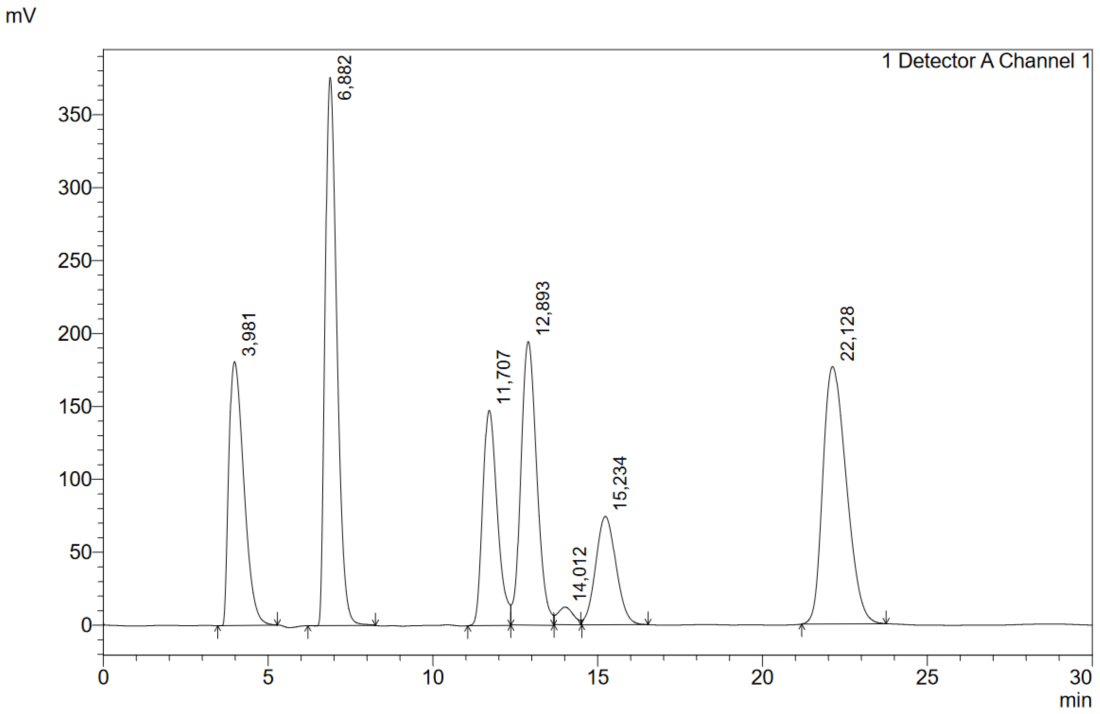

Figure S1 Chromatogram of a standard sample analyzed with the YMC-Pack Polyamine II/S-5 µm/12 nm column. The standard contained water (3.981 min), glycerol (6.882 min), D-fructose (11.707 min), 2-α-D-glucosyl-glycerol (12.893 min), 1-α-D-glucosyl-glycerol (14.012 min), glucose (15.234 min) and sucrose (22.128 min)

In-operando stability of encapsulated LmSucP

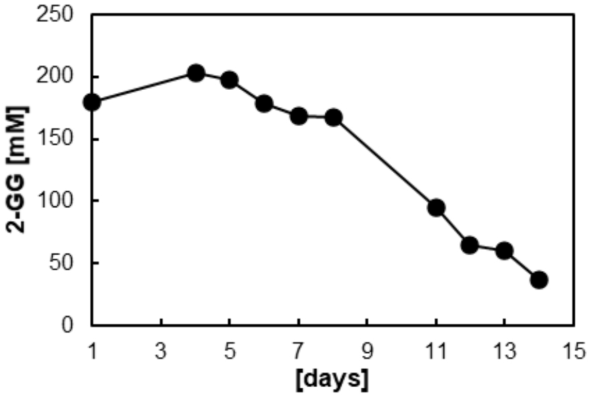

Figure S2 Operando stability of PAM-encapsulated whole cells of *E. coli* expressing *Lm*SucP. Continuous reaction at 30 °C using a packed-bed reactor (25 mL) of PAM-I particles (size ≥2 mm). The space velocity was 0.24 h^{-1}. The 2-α-D-glucosyl-glycerol (2-GG) concentration (●) in the effluent is shown. The PAM-I particles used were prepared with a cell loading of 0.5 g mL^{-1} and an acrylamide concentration of 0.1875 g mL^{-1}

__Packed bed reactors__

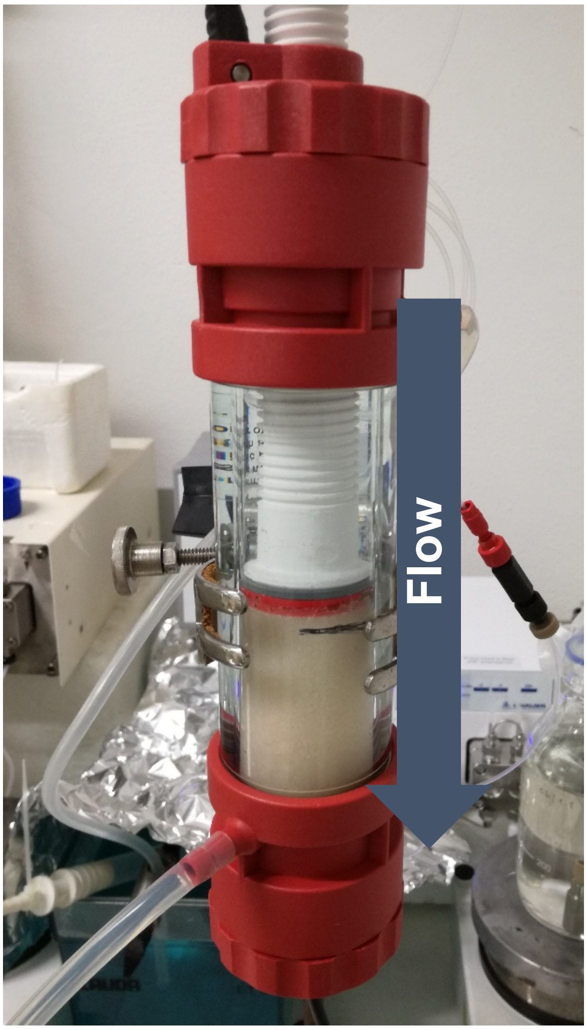

Figure S3 Packed bed reactor, filled with polyacrylamide material (PAM-I), for the continuous production of 2-α-D-glucosyl-glycerol.

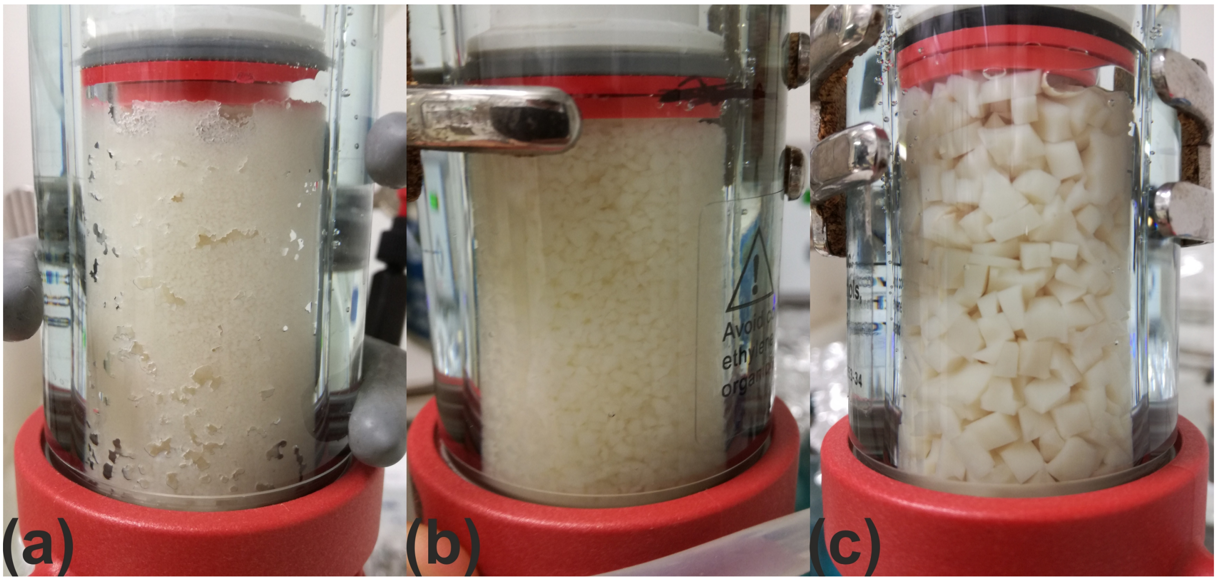

Figure S4 Packings for the continuous 2-α-D-glucosyl-glycerol production. The reactors were filled with PAM-I particles exhibiting a particle size fraction of (a) 0.25-1.00 mm, (b) 1.00-2.00 mm and (c) >2 mm. In (a) the uneven packing, including dead zones, can be seen.

Particle size distribution and dispersity

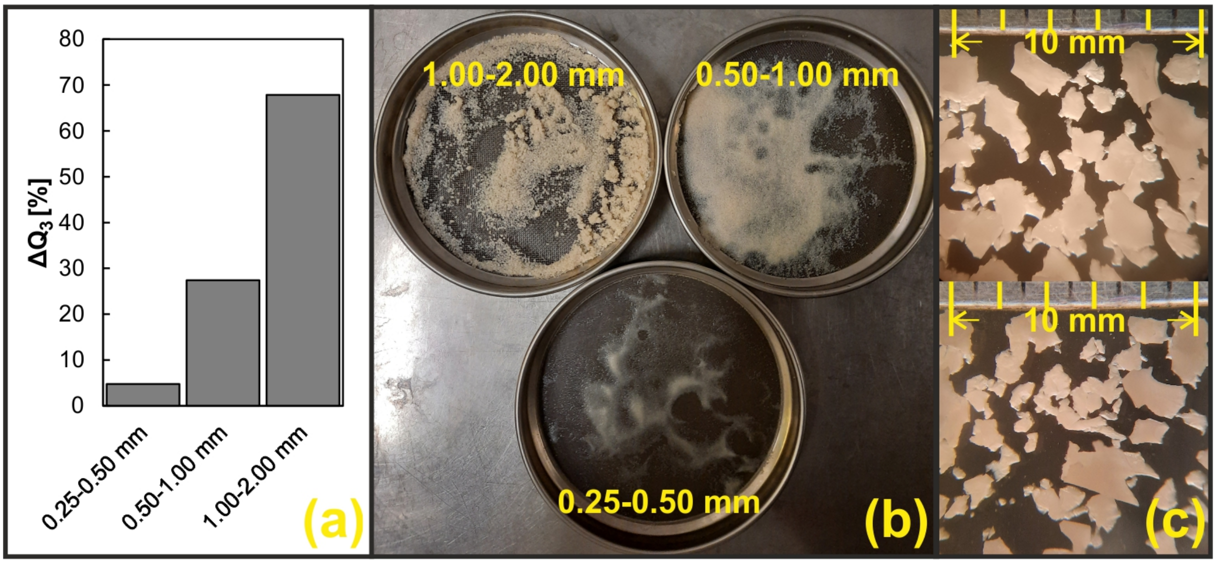

Figure S5 (a) Particle size distribution (PSD, based on mass) of an exemplary PAM-I fraction (0.25-2.00 mm). The PSD was obtained by manual wet sieving using sieves with mesh sizes of 0.25, 0.50 and 1.00 mm. (b) Sieving fractions after the wet sieving. (c) Microscopic images of the PAM-I particles (0.25-2.00 mm)

Comment: the used shredding process is not perfectly controllable and thus particle dispersity and particle size distribution (PSD) may vary from batch to batch. To compensate that to some extent, we included the sieving process in our PAM-I production process (Fig. 1). Nevertheless, a variation of the PSD (shown in Figure S5), within the sieving fraction of 0.25-2.00 mm, cannot be excluded.

References

Franceus J, Ubiparip Z, Beerens K, Desmet T (2021) Engineering of a thermostable biocatalyst for the synthesis of 2-*O*-glucosylglycerol. ChemBioChem cbic.202100192 . https://doi.org/10.1002/cbic.202100192

Goedl C, Sawangwan T, Mueller M, Schwarz A, Nidetzky B (2008) A high-yielding biocatalytic process for the production of 2-*O*-(α-D-glucopyranosyl)-sn-glycerol, a natural osmolyte and useful moisturizing ingredient. Angew Chemie - Int Ed 47:10086–10089 . https://doi.org/10.1002/anie.200803562

Klimacek M, Sigg A, Nidetzky B (2020) On the donor substrate dependence of group-transfer reactions by hydrolytic enzymes: Insight from kinetic analysis of sucrose phosphorylase-catalyzed transglycosylation. Biotechnol Bioeng 117:2933–2943 . https://doi.org/10.1002/bit.27471
